# Assessing point load strength in irregular phyllite via an equivalent area method

**DOI:** 10.1371/journal.pone.0321740

**Published:** 2025-05-14

**Authors:** Hong Zhang, Yong Cao, Qiang Luo, Wei Qi

**Affiliations:** 1 Sichuan Provincial Engineering Research Center of Rail Transit Lines Smart Operation and Maintenance, Chengdu Vocational & Technical College of Industry, Chengdu, China; 2 Key Laboratory of High-Speed Railway Engineering (Ministry of Education), School of Civil Engineering, Southwest Jiaotong University, Chengdu, China; University of Science and Technology Beijing, CHINA

## Abstract

Obtaining intact cylindrical cores from soft, weathered rock is frequently challenging, making the point load test a preferred technique for quickly determining rock strength. This study critically evaluates two widely used point load strength calculations—the loading-span and equivalent-diameter methods—and presents an alternative “equivalent area method” founded on the ratio between the actual failure cross section and the minimum cross section. Irregular phyllite specimens spanning three levels of weathering (heavily, moderately, and slightly) are tested to analyze how shape factor (*β*) and loading span (*D*) affect the point load strength index (*I*_s_). Results show that the area factor has a skewed distribution, with median values increasing from 1.40 to 1.46 as weathering intensifies—substantially exceeding the 0.3 to 1.0 range in the loading-span method and surpassing the 4/π factor used in the equivalent-diameter approach. A recommended median area factor of 1.43 is therefore proposed. The measured *I*_s_ decreases following a power-law trend as *β* and *D* increase, with weathering reducing the sensitivity of *I*_s_ to *β* but not significantly altering its sensitivity to *D*. For heavily, moderately, and slightly weathered samples, the allowable *β* should be at least 0.4, 0.5, and 0.6, respectively, and the loading span should lie between 35 and 80 mm. Unlike the traditional loading-span and equivalent-diameter methods, the proposed equivalent area method incorporates a variable area factor *ψ* that accounts for the actual failure cross section in irregular specimens. This approach reduces scatter in test results and is particularly valuable for soft or weathered rock, where conventional cylindrical core preparation is infeasible. Through extensive testing on phyllite, we demonstrate that this method provides more stable estimates of point load strength and offers practical guidelines for specimen selection, making it highly relevant for geotechnical applications in weak-rock environments.

## 1. Introduction

Uniaxial compressive strength (UCS) is a fundamental parameter in the classification of rock strength and the weathering zonation of rock masses [1]. It is typically obtained from uniaxial compressive tests conducted in the laboratory [2]. Preparing specimens for these tests often involves on-site core drilling, followed by lab-based cutting and polishing, a complex and challenging process. For weak, heavily weathered, or jointed rocks, obtaining intact cores or preparing standard specimens can be difficult [3], complicating UCS measurement through conventional methods.

To address these challenges, the International Society for Rock Mechanics (ISRM) introduced the *Point Load Strength Test* [4]. This method involves applying a concentrated point load to rock specimens between two loading cones, determining the point load strength, and converting it to UCS. Due to its relatively low failure load requirements, the point load apparatus is small, portable, and suitable for both indoor and field tests. Furthermore, specimen preparation is minimal, often requiring only reshaping with a hammer, reducing costs and improving efficiency. As a result, this method is widely used when standard testing is impractical.

Research on the point load strength test can be broadly categorized into three areas: failure mechanisms, computational models, and the factors influencing point load strength and its correlation with compressive and tensile strength [5]. Hiramatsu et al. [6] analyzed the stress distribution in an elastic sphere under point loading, showing that tensile stress dominates, leading to fractures in the central region of the specimen. Reichmuth [7] observed that tensile and compressive zones form under point load, with tensile stress driving failure. Peng [8] performed stress field analysis to confirm the predominance of tensile failure in cylindrical specimens, with limited shear failure near the loading points. These findings were verified through microscopic observations of fracture surfaces.

In 1972, Broch and Franklin [9] introduced a formula to calculate point load strength (*I*_s_), which uses the square of the distance between the loading points as the bearing area. This calculation method was later adopted by the ISRM [4]. Kahraman and Gunaydin [10] refined this approach by proposing a method to calculate *I*_s_ for irregular block specimens using an equivalent core diameter. Other researchers [11] have critiqued the ISRM model, suggesting alternative calculation methods that better reflect specimen failure modes, proposing physically meaningful metrics based on the actual failure area. Several studies have further explored the relationship between point load strength, UCS, and tensile strength. Fu and Wang [12], through tests on various rock types, proposed conversion relationships for UCS and tensile strength with point load strength for radial and axial specimens. Other researchers [13] have reported different conversion factors for these strength parameters, suggesting variability in the UCS to *I*_s(50)_ ratio based on rock type and size effects.

Despite the extensive research and the standardization of testing methods by the ISRM, certain issues remain unresolved, particularly in soft rocks [14]. While the *Point Load Strength Test* has been extensively used for hard rocks, its application in soft rocks is less studied, and the calculation model for *I*_s_ in such rocks requires further refinement. Moreover, there is ongoing debate over whether *I*_s_ should be calculated based on the original distance between the loading points or the distance at failure, with no consensus yet reached [13]. Additionally, for irregular specimens, the shape coefficient and the acceptable range of loading point distances lack standardized guidelines [4,15,16].

This study aims to address these issues by analyzing the discrepancies in existing methods for calculating *I*_s_ and proposing corrections. Specifically, weathered phyllite specimens with varying degrees of weathering were tested using a modified point load apparatus, and the geometric parameters were statistically analyzed to propose reasonable values for irregular phyllite specimens. Furthermore, this study investigates the relationship between point load strength, UCS, and tensile strength, leading to recommendations for specimen geometric dimensions suitable for different weathering stages. Therefore, this study proposes an equivalent area method to address the over- and under-estimation issues of existing calculation models. Specifically, we incorporate a variable area factor *ψ* derived from the ratio of the actual failure cross section to the minimum cross section, thereby addressing specimen irregularities that can significantly affect soft or weathered rocks. This method aims to yield more consistent results, reduce outlier effects, and ultimately provide a clearer framework for practical geotechnical assessments of rock strength.

## 2. Evaluation method for point load strength of irregular specimens

The following subsections delve deeper into how point load strength is quantified for phyllite with irregular geometry. Section 2.1 begins by reviewing two widely adopted computational approaches and their underlying mechanical assumptions, laying the groundwork for the equivalent area method proposed later.

### 2.1 Calculation methods based on loading distance and equivalent diameter

**Fig 1:**
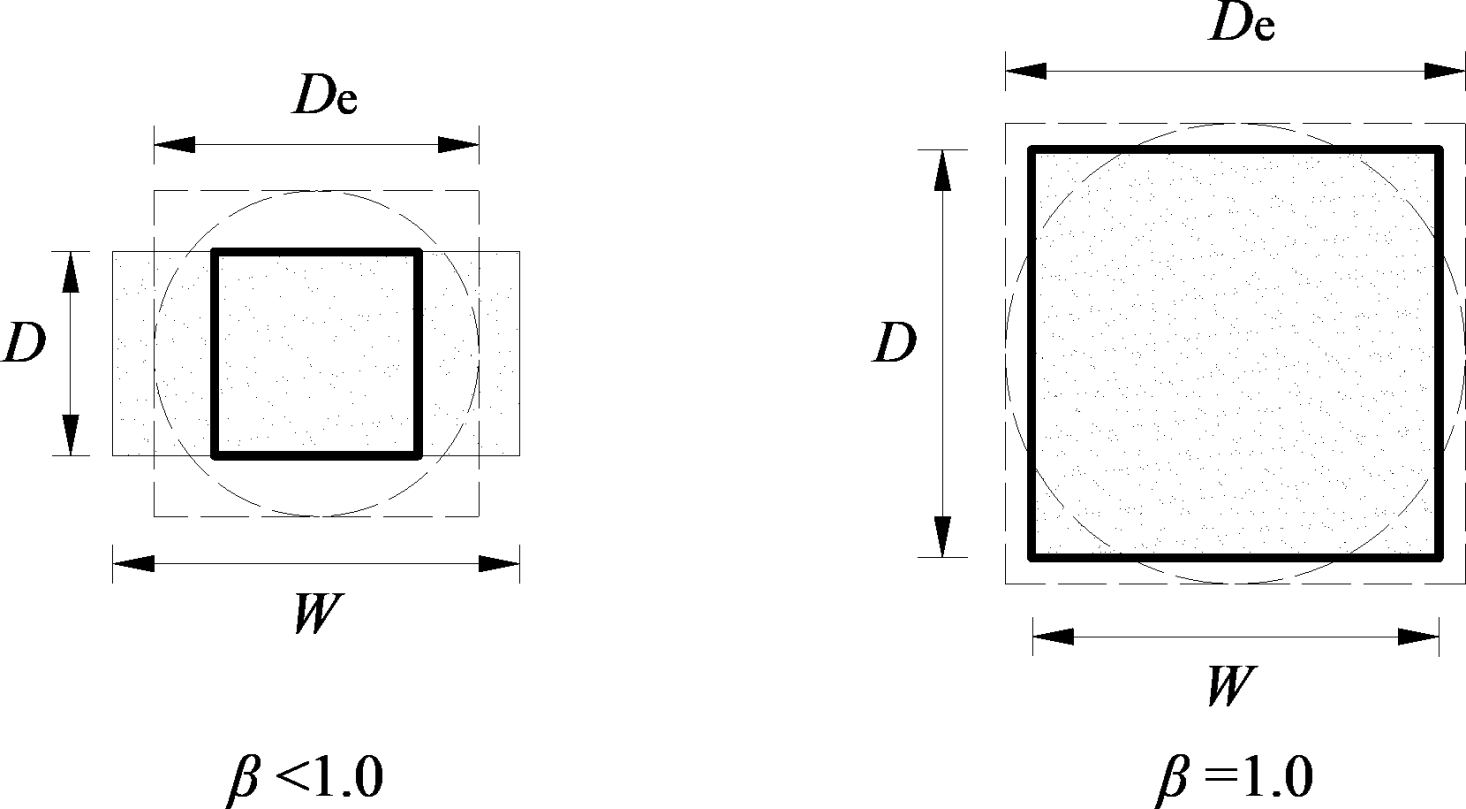
Schematic of calculation for the point load strength.


Research [6–8] demonstrates that under point load, compressive stress is confined to the vicinity of the loading points and a certain depth of the surface, while tensile stress dominates over a larger area, leading to tensile fracture. Therefore, the mechanical essence of point load strength lies in the maximum tensile stress that a specimen can withstand per unit fracture area. For homogeneous, regular cubic specimens, when the shape factor (*β*) and loading point distance (*D*) fall within the specified range, the failure under point load occurs along the minimum cross-section. Here, *β* is defined as the ratio of the loading point distance to the minimum width of the specimen in the orthogonal direction. At this point, the actual area subjected to tensile stress is *WD*, as shown in Fig 1, and the calculation formula for point load strength should be.

$$I_{\text{s}}=P/WD$$ (1)

Regarding the calculation of point load strength, the International Society for Rock Mechanics (ISRM) has proposed two methods. In 1972, the ISRM introduced a definition of the point load strength *I*_s_ as the ratio of the failure load *P* to the square of the distance between the loading points *D*, as shown in Equation (2). Under this definition—often referred to as the “loading-span method”—the tensile stress is assumed to act over an area *D*^2^, as depicted in Fig 1.


$$I_{\text{s}}=P/D^{2}$$ (2)

For cubic or irregular specimens, one may introduce a shape factor β=D/W into Equation (2) to obtain the point load strength. Consequently, for homogeneous specimens, the computed tensile-stress area coincides with the actual load-bearing area only if β=1.0, thereby capturing the true mechanical essence of the point load strength. However, most specimens do not satisfy this condition, in which case the computed area is smaller than the actual load-bearing area (*WD*)—specifically, some multiple of the minimum cross-section—leading to an overestimation of the calculated point load strength.

In 1985, the “loading-span method” was refined through the introduction of an equivalent core diameter *D*_e_. The revised approach, known as the “equivalent-diameter method,” defines the point load strength (*I*_s_) as the ratio of the failure load *P* to the square of the equivalent core diameter *D*_e_, as shown in Equation (3).


$$I_{\text{s}}=P/D_{\text{e}}^{2}$$ (3)

Here, *D*_e_ represents the diameter of a notional circular cross-section having the same area as the specimen’s minimum cross-section, as illustrated in Fig 1. For cubic or irregular specimens, substituting $D_{\text{e}}^{2}=4WD/\pi$ into Equation (3) yields the point load strength. In the case of homogeneous specimens, the computed area exceeds the actual load-bearing area *WD*, specifically by a factor of 4/π relative to the minimum cross-section, thereby causing the calculated point load strength $I_{\text{s}}$ to be underestimated.

### 2.2 Equivalent area method and the unified formula

**Fig 2:**
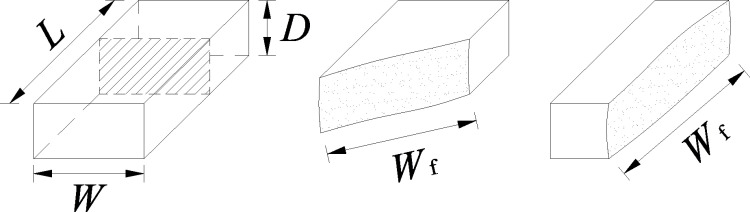
Schematic of the actual failure cross-sectional area.

In point load strength tests, due to the inherent heterogeneity of rock, specimens often fail along surfaces other than their minimum cross section. Drawing on the strength concept, Xiang and Liang [11] examined the theoretical formulation of point load testing and proposed defining *I*_s_ as the ratio of the failure load *P* to the actual failure cross-sectional area *A*_f_, which is given by the product of the failure-section width *W*_f_ and the distance between loading points *D*. This approach—referred to as the “failure-area method”—is illustrated in Fig 2.

By directly measuring the failure cross-sectional area, the “failure-area method” considers how the specific failure pattern of each specimen influences the test result, thus offering a clear mechanical model and a well-defined physical interpretation. In practical applications, it has demonstrated favorable outcomes [11]. However, the test procedure requires measuring *P*, *D*, and *W*_f_ for every specimen. Because specimens may fracture into multiple pieces, determining *W*_f_ can be cumbersome and time-consuming.

To streamline the process, Xiang and Liang [11] introduced an “area factor” (*Ψ*), defined as the ratio of the actual failure cross section to the minimum cross section. This concept underpins an “equivalent area method” and a unified formula (Eq. 4) that utilize the minimum cross section as a basic parameter. The loading-point distance *D* may be taken as the distance (*D*′) at the moment of failure.


$$I_{\text{s}}=P/\Psi WD$$ (4)

Therefore, once *Ψ* has been established for a particular type of rock with similar weathering characteristics, one can measure *W*—the width of the minimum cross section—instead of *W*_f_. Compared with *W*_f_, the parameter *W* is largely unaffected by the failure morphology; it is more stable, more accurate to measure, and especially advantageous when numerous specimens are tested.

From the discussion in Section 2.1, both the “loading-span method” and the “equivalent-diameter method” can be recast as formulations based on the specimen’s minimum cross section. In each case, a specific correction factor—*β* or 4/π, respectively—translates the minimum cross-sectional area into the actual failure cross-sectional area, which is then used to determine *I*_s_. In other words, the “area factor” is *β* in the loading-span method and 4/π in the equivalent-diameter method.

During point load testing, the actual failure cross section is typically larger than the minimum cross section (i.e., *Ψ*>1.0). In the standard [15], the value of *β* is specified between 0.3 and 1.0; consequently, when one adopts *Ψ*=*β* in the loading-span method, the calculated *I*_s_ can exceed that from the “failure-area method” by a factor of two or more.

Although the “equivalent-diameter method” employs a constant *Ψ*=4/π>1.0, consistent with the fact that the failure cross-sectional area generally surpasses the minimum cross-sectional area, it does not account for the influence of rock type or degree of weathering—factors that affect heterogeneity and thus the difference between the failure cross section and the minimum cross section.

By contrast, the “equivalent area method” treats *Ψ* as a variable greater than 1.0, allowing it to accommodate changes in the ratio of the failure cross-sectional area to the minimum cross section that arise from variations in rock properties.

ISRM and AS 4133.4.1 rely on the principle that a rock’s point load strength is calculated from the failure load divided by an idealized area. While the details differ (e.g., in allowable shape factors or permissible specimen size ranges), these standards generally consider either the loading span or an equivalent core diameter to estimate the rock’s cross-sectional area at failure. This works well for more regular geometries but can introduce over- or under-estimates in irregular rock lumps.

Rather than a fixed assumption of how the failure plane relates to the minimum cross section, the proposed method explicitly measures or calibrates an “area factor” (ψ), enabling more direct handling of shapes that do not fail strictly along their smallest cross section or that deviate from standard “ideal” geometries.

Overall, AS 4133.4.1 and similar standards remain valid references for conducting point load tests on irregular rock lumps. However, they do not fully capture the variability in actual failure planes. The proposed equivalent area method addresses this gap by incorporating a variable area factor—offering a more precise, yet still straightforward, solution for soft or weathered rocks where obtaining well-shaped specimens is notoriously difficult.

## 3. Tests and apparatus

Having introduced various methods for evaluating point load strength, we now turn to the experimental program used in this study. Section 3.1 outlines the site conditions, specimen selection process, and the rationale behind choosing phyllite samples with different weathering degrees.

### 3.1 Engineering context and specimen selection

The Menghua Heavy Haul Railway is a dedicated coal transport line that extends from Haolebaoji in Inner Mongolia to Ji’an in Jiangxi Province, with a total length of 1,814.5 km. The segment from Yueyang to Ji’an traverses hilly to low-mountainous terrain, where the stratigraphy predominantly consists of Proterozoic slate, phyllite, and schist, as well as Cretaceous–Tertiary conglomerate, mudstone, and siltstone. These rock types are generally weathered or prone to weathering, often interbedded, and characterized by non-uniform weathering. Along the rail line, a slab-pile retaining structure supports one side of the embankment [17,18]. According to the field investigation, strongly, weakly, and slightly weathered rock layers are encountered at depths of 0.8–7.1 m, 7.1–14.2 m, and 14.2–15.7 m (pile tip), respectively, below the original ground surface.

**Fig 3:**
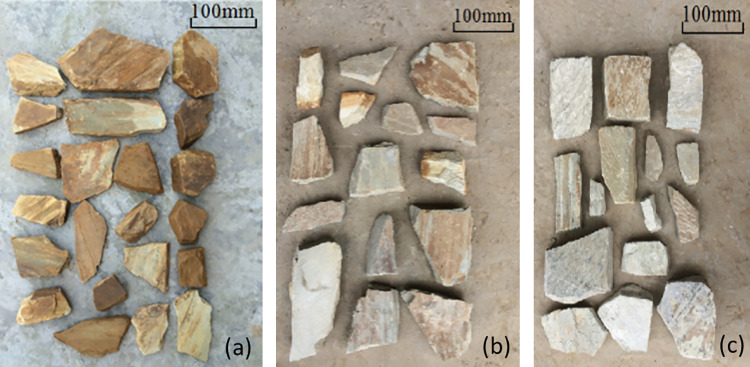
Soft phyllite specimens at different degrees of weathering: (a) strongly weathered; (b) weakly weathered; (c) slightly weathered.

During manual excavation, rock blocks were collected from each respective weathering layer and selected as specimens for point load testing. Each specimen was wrapped in thin plastic film to preserve its natural moisture content [19] and was transported to the laboratory for immediate testing. A subset of the collected specimens is shown in Fig 3.

Weathering grades of phyllite were classified based on widely accepted geotechnical descriptors and visual inspection criteria consistent ISRM standards. Specifically:

- Heavily (Strongly) Weathered: Rock material exhibits pronounced discoloration (yellowish to brown), visible foliation planes are weakened or partially disintegrated, and the strength easily degrades under light hammer blows.
- Moderately Weathered: Rock retains some original structure and color (grayish-yellow), although foliation planes are partially open or softened. It can still exhibit rock-like behavior but shows clear signs of weathering.
- Slightly Weathered: Material is close to fresh color (light gray), with mainly tight foliation planes. Only slight discoloration near rock boundaries and minimal strength reduction.

These classifications were confirmed through site observations and small-scale field tests (e.g., scratch, fracture spacing). Samples were grouped accordingly for laboratory testing.

All point load tests in this study were conducted according to the guidelines outlined in the specification [15], which aligns closely with ISRM recommended methods for point load strength testing. Furthermore, the dimensional tolerances and specimen preparation procedures adhered to ASTM D5731 and AS 4133.4.1, where applicable. The uniaxial compression and Brazilian splitting tests were performed in accordance with ASTM D7012-14 and ASTM D3967-16, respectively.

As the degree of weathering decreases, the phyllite changes gradually from grayish yellow to grayish white. The thickness of selected specimens ranges from 10 to 80 mm, and the value of *β* spans 0.3–1.2. During testing, the loading direction is applied perpendicular to the foliation, hence the specimen’s loading-point spacing *D* can be larger than the width *W* of the minimum cross section. A total of 110, 67, and 69 specimens were tested from the strongly, weakly, and slightly weathered categories, respectively.

**Fig 4:**
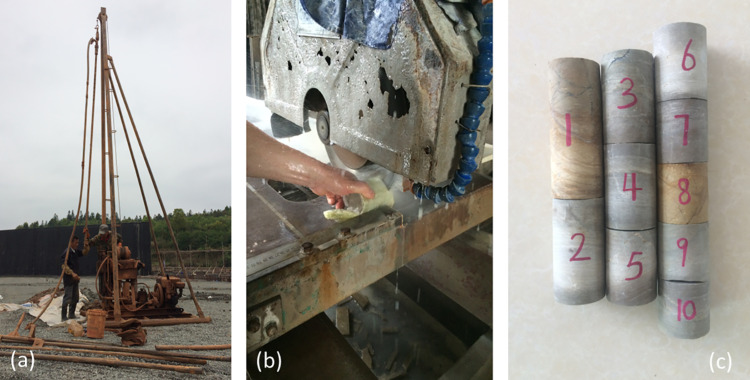
Preparation of uniaxial compression and Brazilian tension test specimens: (a) core drilling; (b) specimen preparation; (c) completed test specimens.

In parallel with the selection of point load specimens, cores of slightly weathered phyllite (diameter 70 mm) were drilled from the same rock layer. Owing to the lower strength of the strongly and weakly weathered phyllite, intact cores could not be obtained for those layers. The slightly weathered cores were cut and ground into specimens for uniaxial compression (Samples #1–#5) and Brazilian splitting tension tests (Samples #6–#10), as shown in Fig 4.

### 3.2 Point load strength testing and instrument modifications

Using a vernier caliper, the specimen length (*L*), the width of the minimum cross section (*W*), and the loading-point spacing (*D*) were measured. The specimen was then placed between the two conical platens of the point load apparatus, with the loading axis perpendicular to the foliation. A gradually increasing load was applied such that failure occurred within 10–60 s. The failure load (*P*) was recorded, and post-failure measurements of the cross-sectional width (*W*) and the loading-point spacing (*D*′) were taken. Only failures in which the failure plane passed through the entire specimen and through both loading points were considered valid. For larger specimens, the remaining fragment from the first test was sometimes retested. In total, 133 data points for strongly weathered, 136 for weakly weathered, and 154 for slightly weathered specimens were deemed valid.

**Fig 5:**
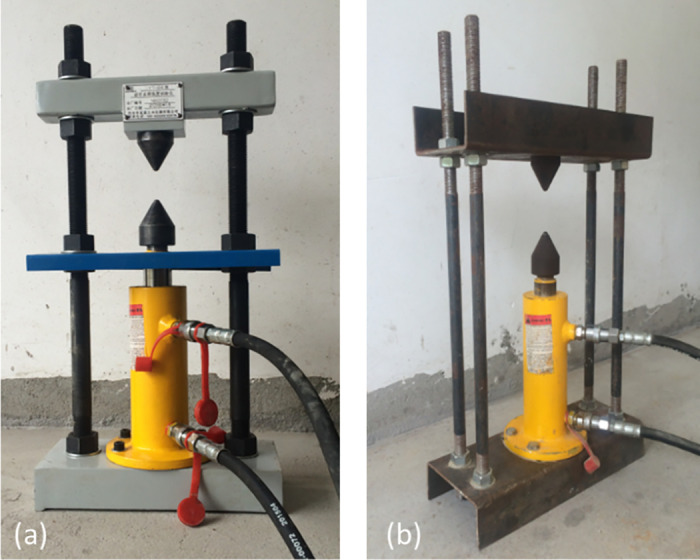
Point load strength apparatus: (a) before modification; (b) after modification.

The point load testing device consists of a loading frame, loading system, and a pressure gauge. Two gauges with ranges of 0–2.5 MPa and 0–25 MPa are available to accommodate rock specimens of varying strengths. Because the conventional single-column frame lacks adequate out-of-plane stiffness, it was replaced with a four-column spatial frame—comprised of top and bottom beams linked by four adjustable vertical columns—to enhance overall rigidity. This modification is illustrated in Fig 5. By employing a four-column spatial frame instead of a single-column design, the apparatus achieves improved lateral rigidity. This reduces off-axis loading and potential specimen misalignment. Preliminary calibration tests (strain gauges) on 10 standard cylindrical samples showed that the new frame reduced lateral deflection by ~40%, thus improving the repeatability of measured failure loads and diminishing the risk of partial, invalid fractures. The enhanced stiffness ensures that the applied force remains more uniformly axial throughout the test.

### 3.3 Uniaxial compression and Brazilian splitting tests

The uniaxial compressive strength *R*_i_ is defined as the load per unit cross-sectional area under unconfined compressive loading, calculated by:


$$R_{\text{i}}=P/A$$ (5)

Here, *P* is the failure load and *A* is the cross-sectional area of the specimen.

For standardized uniaxial compression tests, the cylindrical specimen diameter *d* should be 50±2 mm, with a height-to-diameter ratio (*h*/*d*) of 2.0–2.5, and a loading rate of 0.5 MPa/s. For statistical analysis and comparability, uniaxial compressive strength values *R*_i_ obtained from arbitrarily sized specimens can be converted to a standard strength *R*_c_ using Equations (6)–(8):


$$R_{\text{c}}=K_{\left(h/d\right)}K_{d}R_{\text{i}}$$ (6)

$$K_{\left(h/d\right)}=\frac{8}{7+2\left(d/h\right)}$$ (7)

$$K_{d}={\left(\frac{50}{d}\right)}^{0.18}$$ (8)

Here, $K_{\left(h/d\right)}$ is a shape-effect correction factor, $K_{d}$ is a size-effect correction factor, *d* is the specimen diameter (in mm), and *h* is the specimen height.

Tensile strength *R*_t_ is commonly determined via Brazilian splitting tests on cylindrical specimens loaded diametrically. The recommended specimen diameter is 50±2 mm, with a height-to-diameter ratio of 0.5–1.0 and a loading rate of 0.3 MPa/s. The splitting tensile strength is then calculated by:


$$R_{\text{t}}=2P/\pi dh$$ (9)

## 4. Factor *ψ* and its role

To better represent the actual cross-sectional area involved in specimen failure, this section introduces the area factor (*ψ*). Section 4.1 presents the statistical distribution of *ψ* across different weathering grades, illustrating its practical significance and variability.

### 4.1 Variations in factor *ψ*

**Fig 6:**
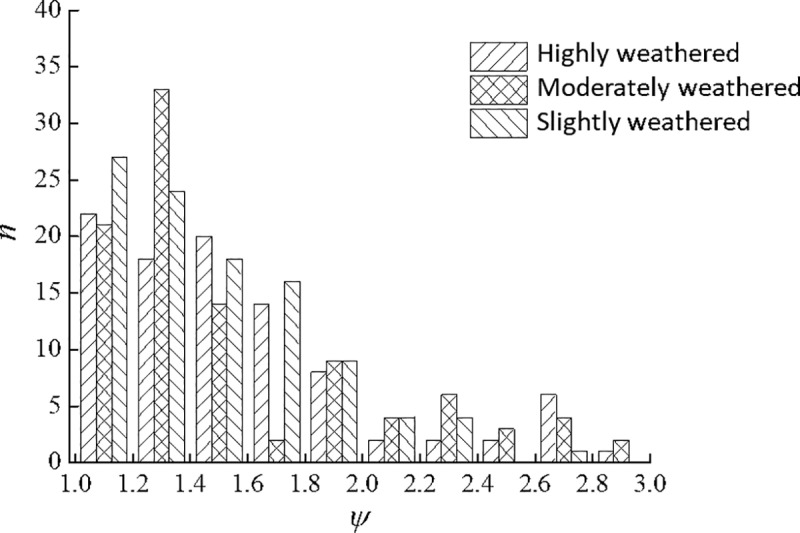
Histogram of the area factorψ.

Drawing on the dimensional requirements for point load test specimens [15], strongly, weakly, and slightly weathered soft phyllite specimens were chosen such that the shape factor *β*=*D*/*W* lies between 0.3 and 1.0, and the loading-point distance *D* between 15 and 100 mm. Here, *D* is the spacing between the loading points, and *W* is the minimum width of the specimen measured orthogonally. Based on measured values of *W*_f_ and *W*, the histogram of the “area factor” *ψ* was constructed, as shown in Fig 6.

Following the ISRM guidelines and national standards [15,16], we selected phyllite specimens such that the shape factor *β*=*D*/*W* lay between 0.3 and 1.0, and the loading-point distance *D* between 15 mm and 100 mm. These ranges ensure valid ‘through-going’ fractures in point load tests and avoid overly large or thin samples that could skew the results or fail in a non-representative manner. Additionally, the upper limit of 100 mm is imposed to reduce partial failure (as recommended by [4]).

From Fig 6, for soft phyllite specimens at different weathering states, *ψ* varies within the range 1.0–3.0. The frequency *n* decreases sharply as *ψ* increases, indicating a distinctly skewed distribution. To provide a quantitative measure of distribution symmetry, one typically employs the skewness coefficient *S*_*K*_ [20], defined by:


$$S_{K}=\frac{n\sum_{i=1}^{n}{\left(x_{i}-\bar{x}\right)}^{3}}{\left(n-1\right)\left(n-2\right)S^{3}}$$ (10)

Here, *n* is the number of samples, $\bar{x}$ is the sample mean, and *S* is the sample standard deviation. A perfectly symmetrical distribution yields $S_{K}=0$. A positive $S_{K}$ implies a right-skewed distribution, whereas a negative $S_{K}$ signifies a left-skewed distribution. If $\left|S_{K}\right|$ >1.0, the data show highly skewed behavior; if $0.5\leq \left|S_{K}\right|\leq 1.0$, the skewness is considered moderate; and if $\left|S_{K}\right|\leq 0.5$, the skewness is small [20].

The calculated $S_{K}$ values for strongly, weakly, and slightly weathered specimens are 1.16, 1.18, and 1.12, respectively, indicating a high level of skewness. In such cases, the arithmetic mean is sensitive to extreme values and does not represent the overall data well. Consequently, the median often serves as a more robust measure [20]. The median values of *ψ* for strongly, weakly, and slightly weathered specimens are 1.46, 1.43, and 1.40, respectively, showing a slight decrease with lower degrees of weathering. This observation implies that the actual failure cross-sectional area becomes increasingly similar to the minimum cross-sectional area as weathering weakens—an indication that the rock becomes more uniform. For soft phyllite, when using the “equivalent area method” to calculate *I*s, it is recommended to set the area factor *ψ* to an average value of approximately 1.43.

It should be emphasized that the median *ψ*≈1.43 reported here is specific to phyllite under the tested conditions (i.e., degree of weathering, anisotropy, and specimen geometry). For other rock types or different degrees of heterogeneity, the median *ψ* may differ. We recommend performing a preliminary calibration on a small batch of samples whenever possible to establish a representative *ψ* for that particular material.

Owing to the high skewness (*S*_K_ ≈1.12–1.18) of the *ψ* distribution, the median *ψ* is a more robust central value than the mean, as it mitigates the impact of extreme outliers. This leads to more reliable strength estimates when using the equivalent area method, given that occasional specimens might fail along atypical planes. The method’s reliability thus benefits from focusing on median-based parameters instead of mean-based metrics when defining *ψ*.

### 4.2 Applicability of the “equivalent area method” for calculating the point load strength

To evaluate the suitability of the “equivalent area method” for calculating point load strength, strongly, weakly, and slightly weathered soft phyllite specimens with *β* between 0.3 and 1.0 and a loading-point distance between 15 and 100 mm were selected from the test dataset. Three calculation approaches were applied—namely, the “loading-span method,” the “equivalent-diameter method,” and the “equivalent area method”—resulting in 106, 110, and 112 valid specimens for each approach, respectively. The corresponding mean *I*s values, standard deviations *S*, and coefficients of variation *δ* are summarized in Table 1.

**Table 1:** Statistical parameters from three calculation methods.

Methods	Degree of weathering	$\bar{I_{s}}$ /MPa	*S*	*δ*
Loading-span	Highly	0.850	0.473	0.557
	Moderately	2.003	1.130	0.564
	Slightly	3.883	1.785	0.460
Equivalent-diameter	Highly	0.403	0.168	0.417
	Moderately	0.979	0.401	0.410
	Slightly	1.692	0.478	0.282
Equivalent area	Highly	0.314	0.121	0.385
	Moderately	0.761	0.290	0.381
	Slightly	1.290	0.332	0.258

As indicated in Table 1, for heterogeneous specimens at different weathering levels, the “loading-span method” yields the largest *S* and *δ*, reflecting the greatest variability in the results. Furthermore, its calculated Is exceeds that derived via the “equivalent area method” by more than a factor of two, suggesting that the assumed tensile area is much smaller than the actual load-bearing area. In contrast, the “equivalent-diameter method” produces relatively lower S and δ values; its *I*s is closer to that from the “equivalent area method,” implying that the computed tensile area is more realistic. Among the three methods, the “equivalent area method” yields the smallest *S* and *δ*, indicating the least data scatter and the most stable test results. Hence, the “equivalent area method” is recommended for calculating the point load strength of these soft phyllite specimens.

## 5. Key factors influencing the point load strength

Recognizing that multiple variables can influence the measured point load strength, we next examine how specimen geometry and testing conditions alter the outcomes. Section 5.1 specifically investigates the penetration depth (Δ*D*) and its effect on the point load strength (*I*_s_).

### 5.1 Effect of penetration depth Δ*D* on *I*_s_

**Fig 7:**
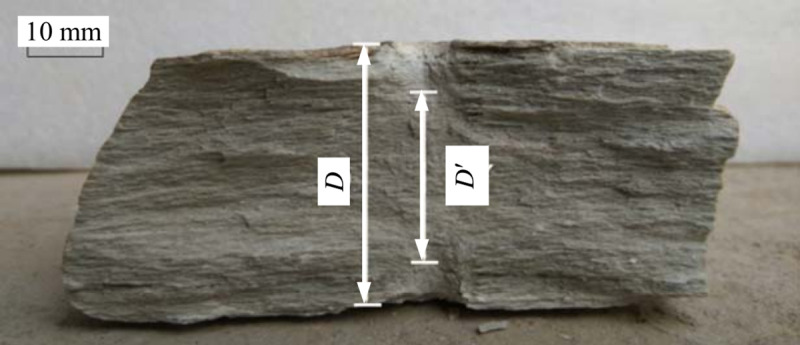
Schematic for measuring *D* and *D*′ values.

During testing, the conical platens penetrate the specimen to a certain depth, reducing the final loading-point distance at failure (*D*′) relative to the initial distance (*D*). Experimental results indicate that the penetration depth Δ*D*=*D*−*D*′ can be significant and may noticeably affect the test outcomes. Consequently, it is worthwhile to examine the variation of Is with Δ*D*. Defining *α*=Δ*D*/*D* as the “penetration-depth coefficient,” one obtains *D*′ by measuring the indents in the specimen’s cross section immediately after failure, as illustrated in Fig 7.

Referring to Equation (4), one can use the “equivalent area method” to compute *I*s. When the loading-point distance is taken as *D* versus *D*′, then the relative deviation *w*_1_ = Δ*D*/ *D*′.

**Fig 8:**
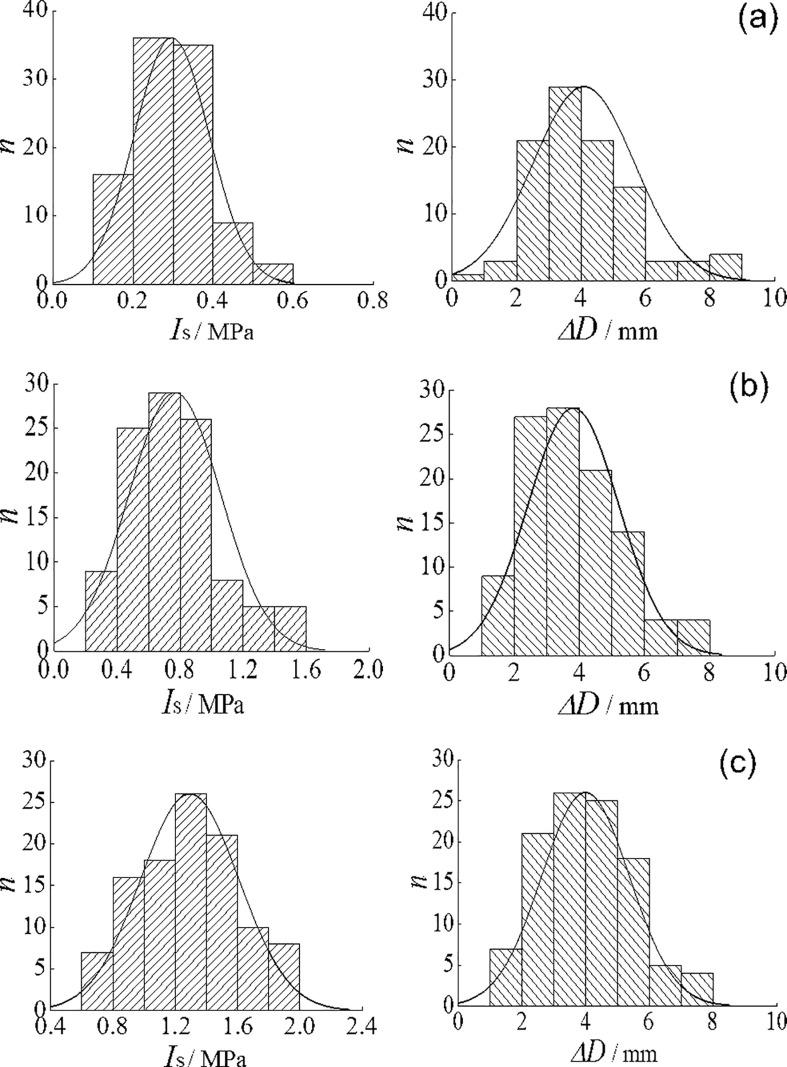
Histograms of *I*_s_ and Δ*D.* (a) strongly weathered; (b) weakly weathered; (c) slightly weathered.

In analyzing the effect of Δ*D* on *I*s, specimens were again selected such that *β* lies between 0.3 and 1.0 and *D* between 15 and 100 mm [15]. A total of 106, 110, and 112 specimens were considered for the strongly, weakly, and slightly weathered categories, respectively. To improve the reliability of the results, outliers were systematically removed based on the “3σ” criterion: (1) any anomalous *I*_s_ values were discarded along with their corresponding specimens, and (2) any anomalous Δ*D* values were also eliminated. These steps were repeated until all remaining Is and Δ*D* values satisfied the normal distribution assumption. In the end, 99, 107, and 106 valid specimens remained for the strongly, weakly, and slightly weathered categories, respectively. Fig 8 presents the histograms of *I*_s_ and Δ*D* computed via Equation (4), and Table 2 summarizes the ranges, means, and standard deviations for *I*_s_, Δ*D*, and *α*.

**Table 2:** Statistical results for *I*_s_, Δ*D*, and *α* after outlier removal.

Indicators	Degree of weathering	Range	Mean	*S*
*I*_s_/MPa	Highly	0.115−0.562	0.297	0.094
	Moderately	0.336−1.532	0.767	0.292
	Slightly	0.658−1.915	1.293	0.316
Δ*D*/mm	Highly	0.63−8.62	4.08	1.579
	Moderately	1.24−7.78	3.81	1.401
	Slightly	1.49−7.58	4.00	1.383
*α*/%	Highly	2.57−27.28	10.86	5.038
	Moderately	4.32−29.84	11.81	4.946
	Slightly	4.63−29.11	13.75	5.420

The test data reveal that *I*_s_ increases progressively from strongly to weakly to slightly weathered specimens, while Δ*D* remains relatively consistent at about 3.81–4.08 mm. The penetration-depth coefficient *α* lies in the range of 10.86%–13.75% and shows a modest increase as *I*_s_ rises—i.e., as the weathering weakens—consistent with the findings [16]. The resulting *w*_1_ for specimens with different weathering degrees spans 12.18%–15.94%, indicating that Δ*D* has a non-negligible effect on *I*_s_. When precise strength measurements of soft phyllite are required, the influence of penetration depth should thus be accounted for.

In summary, when examining the impact of *β* and *D* on the point load strength *I*_s_, one should employ the distance D′ at failure in Equation (4) for more accurate results. In addition, the area factor *ψ* is best taken as its average value (1.43), as previously discussed.

### 5.2 Effect of shape factor β on *I*_s_

When conducting point load tests on irregular specimens, both the shape factor *β* and the loading-point distance *D* can strongly influence the results. Ideally, the relationship among *β*, *D*, and the point load strength *I*_s_ should be investigated by referencing a baseline specimen with *D*=50 mm and *β*=1.0.

**Fig 9:**
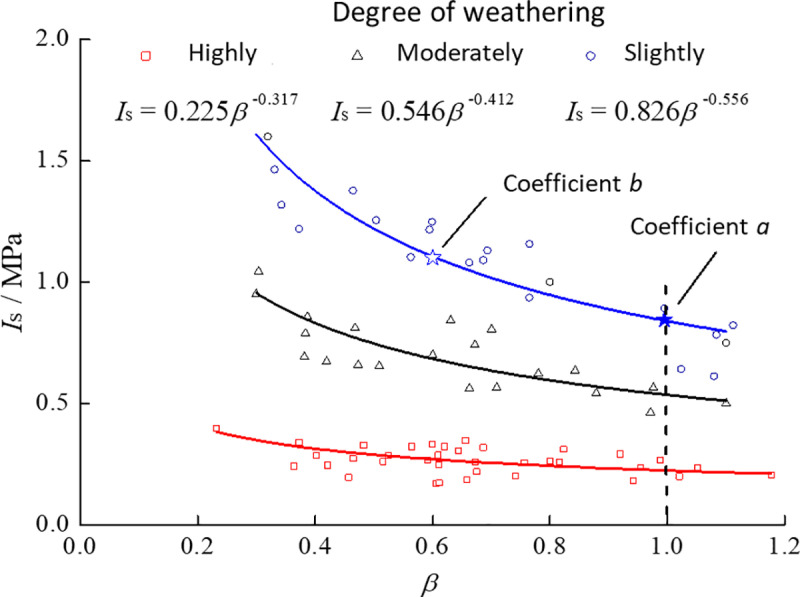
Relationship between *I*_s_ and *β* at different weathering levels (R^2^: 0.88, 0.83, 0.91 for highly, moderately, and slightly).

In studying the relationship between *I*_s_ and *β*, the specimen’s loading-point distance *D* should be held constant at 50 mm to eliminate size effects. However, because the specimens are irregular, only a few satisfy *D*=50 mm. When 44 mm≤*D*≤59 mm, the relative deviation in *I*_s_ [Equation (4)] compared with a reference specimen having *D*=50 mm remains below 15%. Therefore, small variations in *D* within this range can be neglected. Consequently, specimens with *D* in the 44–59 mm range were selected, and the relationship between *I*_s_ and *β* was plotted separately for strongly, weakly, and slightly weathered specimens, as shown in Fig 9.

To minimize size effects in assessing the relationship between *I*_s_ and *β*, only specimens with 44≤D≤59 mm were used. This subset had fewer total samples than our entire dataset, but it allowed more consistent comparisons. When we repeated the same analysis with all specimens (i.e., 15≤D≤100 mm and 0.3≤*β*≤1.2), we observed the same overall decreasing trend of *I*_s_ with *β*, albeit with slightly higher scatter (coefficient of variation increased by roughly 10–15%). Hence, restricting *D* to 44–59 mm helps control for size effects and yields more stable curve-fitting results.

From Fig 9, for specimens at different weathering levels, *I*_s_ decreases with increasing *β*, eventually stabilizing; this trend is well described by a power function. The fitted power exponents for strongly, weakly, and slightly weathered specimens are −0.556, −0.412, and −0.317, respectively. This finding indicates that *β* has a greater effect on *I*_s_ for rocks that are less weathered. Taking the *I*_s_ value at *β*=1.0 as a reference, i.e., coefficient *a*, and the theoretical value from the fitted function as the coefficient *b* (Fig 9), the relative deviation *w*_2_ is defined as:


$$w_{2}=\frac{\left|b-a\right|}{a}\times 100\%$$ (11)

Because *I*_s_ follows a power-law decrease with increasing *β*, a “critical shape factor” *β*′ arises under various allowable relative deviations. When *β*≥*β*′, the relative deviation remains within the specified limit; otherwise, it exceeds that threshold. Table 3 shows the values of *β*′ for specimens at different weathering levels.

**Table 3:** Values of critical shape factor (*β*′) for different allowable relative deviations (*w*_2_, in %).

Weathering level	10%	20%	30%	40%	50%
Highly	0.74	0.56	0.44	0.35	0.28
Moderately	0.79	0.64	0.53	0.44	0.37
Slightly	0.84	0.72	0.62	0.54	0.48

Regarding permissible *β*-value ranges, various guidelines [16] recommend *β* between 0.3 and 1.0 or between 0.5 and 1.0; Ref. [15] suggests 0.3–1.0 and notes an optimal range of 0.75–1.0. For soft phyllite, *β*=0.3 can lead to relative deviations as high as 95.3% in slightly weathered specimens—clearly too large. Although approaching *β* closer to 1.0 reduces data scatter, it also restricts specimen selection and hence limits the use of point load tests. Given the strong heterogeneity and anisotropy of rock materials, data scatter is typically high. Thus, choosing *β*′ to satisfy *w*_2_ =40% is recommended as the practical criterion for specimen selection. Moreover, because the influence of *β* on *I*_s_ varies with weathering degree, the recommended *β*-value range should differ for each weathering level. In conjunction with the test results, the suggested range of *β* is 0.4–1.0, 0.5–1.0, and 0.6–1.0 for strongly, weakly, and slightly weathered specimens, respectively.

### 5.3 Effect of the loading-point spacing *D* on *I*_s_

**Fig 10:**
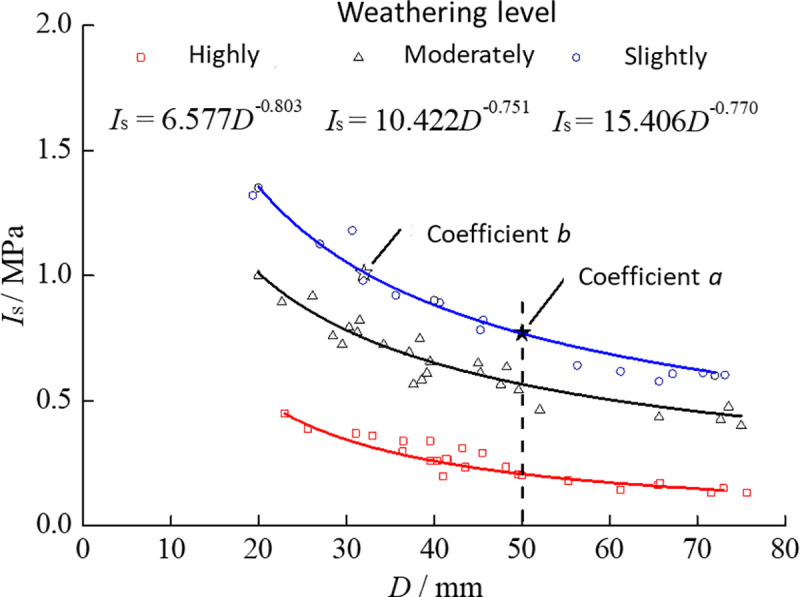
Relationship between *D* on *I*_s_ at different weathering levels (R^2^: 0.82, 0.89, 0.93 for highly, moderately, and slightly).

When investigating size effects, one ideally sets the shape factor *β*=1.0, i.e., *D*/*W*=1.0. However, due to the irregular geometry of the specimens, achieving exactly *β*=1.0 is often impractical. From previous analysis, once *β*≥0.6, even the most sensitive (slightly weathered) specimens exhibit stable strength. Therefore, in examining how *D* influences *I*_s_, specimens with 0.8≤*β*≤1.2 were selected so that variations in shape factor could be neglected. Fig 10 depicts the relationship between *I*_s_ and *D* for specimens at varying degrees of weathering.

Similar to the shape-factor analysis, restricting *β* to the 0.8–1.2 range reduces the total sample size but ensures minimal shape effects. If all specimens are included, the same overall power-law behavior emerges, but with a larger scatter in *I*_s_ (the coefficient of variation increases by approximately 8–12%). Thus, focusing on specimens near *β*≈1.0 isolates the size effect of *D*. The fitted power-law exponents for strongly, moderately, and slightly weathered phyllite are −0.80, −0.75, and −0.77, respectively, with corresponding R^2^ values of 0.82–0.93 (Fig 10).

As shown in Fig 10, the overall effect of *D* on *I*_s_ is essentially similar to that of *β* on *I*_s_. It can also be described by a power function. The fitted power exponents for strongly, weakly, and slightly weathered specimens are −0.803, −0.751, and −0.770, respectively, with only minor differences among them. Likewise, the similar shapes of the curves suggest that the influence of *D* on *I*_s_ is not strongly dependent on weathering degree. Taking *D*=50 mm as the reference, one can calculate the permissible ranges of *D* at different relative deviations. The results for specimens with varying weathering degrees are presented in Table 4.

**Table 4:** Specimen *D* ranges at different allowable relative deviations (*w*_2_, in %).

Weathering level	10%	20%	30%	40%	50%
Highly	45−57	40−66	36−78	33−95	30−119
Moderately	44−58	39−67	35−81	32−99	29−126
Slightly	44−57	40−67	36−80	32−97	30−123

Different standards [15,16] specify different acceptable intervals for *D*, with the narrowest range being 30–50 mm and the broadest 15–100 mm. Previous research [4] indicates that for specimens with *β*=1.0, if *D*>100 mm, partial failure occurs in 35%–52% of tests, preventing the acquisition of valid *I*_s_ values. In the present study, the maximum *D* measured was 72.31 mm; therefore, an upper limit of 80 mm is reasonable. For soft phyllite, the effect of *D* on *I*_s_ does not strongly correlate with weathering degree. Using the same criterion of ω2=40%\omega_2 = 40\%ω2 =40% and considering the test results, *D* can be taken between 35 mm and 80 mm for strongly, weakly, and slightly weathered phyllite, aligning closely with the 30–80 mm range [4].

In summary, for the tested specimens, *I*_s_ decreases gradually in a power-law fashion as both *β* and *D* increase, eventually stabilizing. This outcome is consistent with previous findings derived from regular, hard-rock specimens [4]. Meanwhile, in the irregular, soft-rock specimens used here, as the degree of weathering intensifies, the influence of *β* diminishes and the effect of *D* changes only marginally.

### 5.4 Relationship between point load strength and compressive/tensile strength

**Fig 11:**
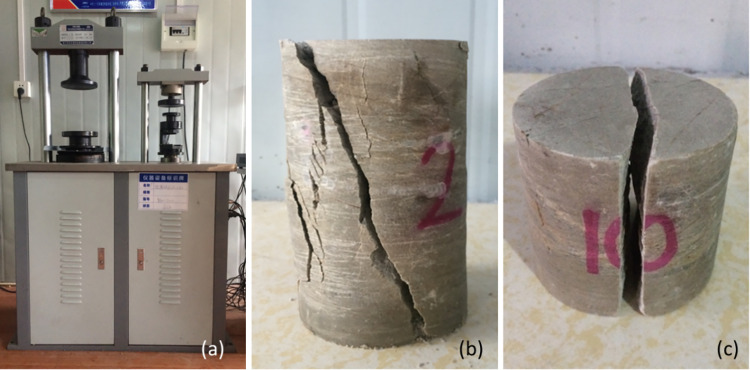
Testing equipment and typical specimen failure modes: **(a)** Compression testing machine; **(b)** Compression test specimen; **(c)** Tensile test specimen0.

While studying the correction methods for calculating point load strength and its influencing factors, several cores of slightly weathered phyllite were obtained from the corresponding rock layer. These cores were then tested under compression and tension to preliminarily explore the relationships among *R*_c_, *R*_t_, and *I*_s_. Uniaxial compressive and Brazilian splitting tests were conducted using a computer-controlled, servo-hydraulic testing machine. Typical failure modes of the specimens are shown in Fig 11, and the specimen dimensions and corrected test results appear in Table 5.

**Table 5:** Dimensions and test results for compressive and tensile strength specimens.

Test	ID	*h*/mm	*d*/mm	*R*_c_/MPa	$\bar{R_{\text{c}}}$/MPa
Uniaxial Compression	KY-01	162.89	70.16	23.0	30.9
	KY-02	125.23	70.54	32.1	
	KY-03	106.96	70.48	28.4	
	KY-04	102.41	70.61	34.2	
	KY-05	89.28	70.37	28.8	
Splitting	KL-01	85.98	70.44	2.6	2.7
	KL-02	74.13	70.51	2.7	
	KL-03	72.19	69.49	1.4	
	KL-04	70.59	70.55	3.2	
	KL-05	65.58	70.66	2.9	

According to [16], if the difference between the maximum and minimum values exceeds 20% of the mean, the data should be discarded. Based on the remaining data, the average uniaxial compressive and tensile strengths of the slightly weathered samples were determined to be 30.9 MPa and 2.7 MPa, respectively, yielding a ratio of 11.4. As such, these samples can be characterized as a brittle material with a uniaxial compressive to splitting tensile strength ratio greater than 8, suitable for point load strength testing [21]. From Table 2, *I*_s_ of the slightly weathered specimens was 1.293 MPa, implying a compressive-strength ratio of 23.9, which is close to 23.7 [9]. The tensile-strength ratio was 2.1, falling within the 1.5–3.0 range specified in [16].

For strongly and weakly weathered soft phyllite, the compressive and punching (shearing) effects under point loading may increase but do not alter the primary tensile-stress regime occurring within the specimen. On the other hand, even though the theoretical “point” contact may differ somewhat from the actual contact condition, as long as repeatable and stable test results can be obtained, point load strength testing remains applicable to soft rock [9].

To statistically validate the differences in variance, we performed an F-test comparing the equivalent area method with the loading-span method. One representative case was employed. For heavily weathered specimens (*n*=50), the computed F-ratio was 1.95 (p = 0.032), indicating significantly lower variance in *I*_s_ when using the equivalent area approach. Similar trends were observed for moderately and slightly weathered groups (F-ratios of 2.10 and 1.81, respectively, both p < 0.05), confirming that the equivalent area method consistently yields lower variance (i.e., more stable results).

## 6. Conclusions

This study introduces a novel equivalent area method for point load testing that explicitly incorporates a variable area factor *ψ*, thereby capturing the actual failure cross-sectional area in irregular specimens. Compared to both the loading-span and equivalent-diameter methods, the new approach significantly reduces data scatter, particularly for soft or weathered phyllite. By evaluating a wide range of specimen geometries and weathering levels, we establish practical guidelines that ensure reliable results: namely, limiting *β*≥0.4–0.6 (depending on weathering degree) and 35≤*D*≤80 mm. These thresholds balance test validity against the realities of field sampling, where obtaining perfectly shaped cores is often infeasible.

Furthermore, the proposed method’s core innovation—the recognition and quantification of a larger failure plane compared to the minimum cross section—accounts for rock heterogeneities and fissures that typical procedures tend to overlook. Our statistical comparisons and F-tests confirm that adopting *ψ* as an adjustable factor yields less variability in point load strength estimates. In addition, we demonstrate strong correlations between the measured point load strength and uniaxial compressive or tensile strengths for slightly weathered phyllite, underscoring the broader applicability of this method. These findings not only extend the practical utility of point load tests to weaker rock formations but also contribute fresh insights into how specimen geometry, weathering, and anisotropy govern fracture behavior under concentrated loading. Future research could apply this framework to other rock types, further calibrating *ψ* and advancing our collective understanding of rock strength characterization.

## Supporting information

S1 Table Raw dataset Fig. 6. (XLSX)

S2 File Inclusivity in global research. (DOCX)
